# Development and validation of a novel nomogram to predict overall survival in gastric cancer with lymph node metastasis

**DOI:** 10.7150/ijbs.39161

**Published:** 2020-02-10

**Authors:** Minjie Mao, Ao Zhang, Yi He, Lin Zhang, Wen Liu, Yiling Song, Shuqi Chen, Guanmin Jiang, Xueping Wang

**Affiliations:** 1 Department of Laboratory Medicine, State Key Laboratory of Oncology in South China, Collaborative Innovation Center for Cancer Medicine, Sun Yat-sen University Cancer Center, Guangzhou, China; Sun Yat-sen University Cancer Center, Guangzhou, China; 2 State Key Laboratory of Oncology in South China, Collaborative Innovation Center for Cancer Medicine, Sun Yat-sen University Cancer Center, Guangzhou, China; 3 Tianjin Medical University Cancer Institute and Hospital, National Clinical Research Center for Cancer, Tianjin's Clinical Research Center for Cancer, Key Laboratory of Cancer Prevention and Therapy, Tianjin, China; 4 Guangzhou Medical University, Guangzhou, China; 5 Department of Clinical Laboratory, The Fifth Affiliated Hospital, Sun Yat-sen University, Zhuhai, Guangdong, China

**Keywords:** gastric cancer, prognosis, nomogram, lymph node metastasis

## Abstract

Gastric cancer (GC) with lymph node metastasis (LNM) at diagnosis is associated with a unstable prognosis and indefinite survival times. The aim of the present study was to construct and validate a model for the Overall survival (OS) estimation for patients with LNM. The nomogram was constructed to predict the OS for LNM-positive GC using the primary group of 836 patients and validated using an independent cohort of 411 patients. Factors in the nomogram were identified by multivariate Cox hazard analysis. The predictive capability of nomogram was evaluated by calibration analysis and decision curve analysis. Multivariate analysis suggested that eight pre-treatment characteristics were used for developing the nomogram. In the primary cohort, the C-index for OS prediction was 0.788 (95% CI: 0.753-0.823), while in validation cohort, the C-index for OS prediction was 0.769 (95% CI: 0. 720-0.818). The calibration plot for the probability of OS and decision curve analyses showed an optimal agreement. Based on the nomogram, we could divided patients into three groups: low-risk group, middle-risk group and a high-risk group(p <0.001).Taken together, we have provided an easy-to-used and accurate tool for predicting OS, furthermore could be used for risk stratification of OS of LNM-positive GC patients.

## Introduction

Gastric cancer (GC) is one of the leading causes of cancer mortality worldwide with about one million new cases reported every year, posing a burden to patients across the globe 1-3. Lymph node metastasis (LNM) is the most common metastasis form of GC and the high mortality rate in GC is mostly influenced by formation of LNM. Therefore, LNM-positive GC patients are often nonresectable in advanced stage at the time of diagnosis, and is generally associated with a poor prognosis 4. Lymph nodes are invaded by tumor cells to influence the immune system, which in turn is closely related to inflammation, and inflammation promotes tumor metastasis 5. Recently, a novel systemic immune-inflammation index (SII) was developed and has proved to be a powerful prognostic indicator of poor outcome for hepatocellular carcinoma patients and colorectal cancer 6. Besides, coagulation pathway is essential for the establishment of metastasis in experimental model systems. High serum LDH has been shown reported in previous studies to confer prognostic information in lymphoma 7, multiple myeloma 8, and small cell lung cancer 9. Therefore, LNM-positive GC comprises a heterogeneous group of patients, and variation in inflammation, metabolism of tumor, blood routine examination and coagulation, we should pay more attention to accurate assessment.

Reliable prognostic information is desired for monitor of individual patients with LNM-positive GC. The most extensive staging system for GC is the Union for International Cancer Control (UICC)/American Joint Committee on Cancer (AJCC) tumor, lymph node and metastases (TNM) staging system^3^,^10^. According to the depth of primary tumor invasion (T stage), regional lymph node metastasis (N stage) and distant metastasis (M stage), the TNM staging system divides patients with GC into different stages 11. TNM staging system ignores the biological heterogeneity of LNM-positive GC patients and it is inadequate for predicting recurrence, which cause large variations in the clinical practice even patients with similar treatment strategies 12,13. Thus, some other factors should be considered for as a new prognostic nomogram for LNM-positive GC, such as SII based on lymphocyte counts, platelet counts, and neutrophil counts, tumor metabolism including LDH, and biomarker of GC including CA199, CEA.

The aim of this study was to generate and internally validate a nomogram based on widely available pretreatment clinical and laboratory data to improve our ability in predicting survival in LNM-positive GC patients.

## Methods

### Patient selection

A total of 1247 GC patients with Lymph node metastasis admitted to Sun Yat-sen University Cancer Center (SYSUCC, Guangdong, China) between December 2010 and July 2017 were enrolled. We randomly allocated the patients into two cohorts: primary cohort and validation cohort. All the patients were classified as the first record of hospitalizations and the clinical information and serum biomarkers were extracted from Electronic Medical Record (EMR) system and Laboratory Information System (LIS). The inclusion criteria were as follows: (1) patients with a confirmed histologically diagnosed of GC; (2) patients with Lymph node metastasis positive; (3) patients without second tumor, or indefinite diagnoses; (4) patients with complete clinical data; (5) patients without concomitant diseases associated with influenced plasma coagulation levels (i.e., VTE, pulmonary embolism, or disseminated intravascular coagulation (DIC) within 1 month of study onset or during the subsequent treatment); (6) patients who not regularly took procoagulant or anticoagulant therapy or took blood transfusions within 1 month of study onset. All patients provided written informed consent. The Institute Research Ethics Committee of the Sun Yat-Sen University Cancer Center, Guangzhou, China approved this study. It was conducted in accordance with the ethical standards of the World Medical Association Declaration of Helsinki. The authenticity of this article has been validated by uploading the key raw data onto the Research Data Deposit public platform (www.researchdata.org.cn), with the approval RDD number as RDDA2020001396.

### Predictor variables

The following variables of interest were collected for each patient: age, sex, TNM stage, Tumor stage, Metastasis, Lauren, platelet-lymphocyte ratio (PLR), neutrophil-lymphocyte ratio (NLR), (lymphocyte-mononuclyte)LMR, lactate dehydrogenase (LDH), prothrombin time (PT), activated partial thromboplastin time (APTT), thrombin time (TT), fibrinogen (Fbg), carcinoembryonic antigen (CEA), carbohydrate antigen 19-9 (CA199). Statistical analyses were performed using SPSS 16.0 (IBM, Chicago, IL, USA). The optimal cut-off points in our study were evaluated by minimum P value from log-rank ×2 statistics using the X-tile program 14 and continuous variables were transformed to categorical variables, while the categorical variables were classified based on clinical findings. Univariate and multivariate regression analysis was used to analyze the risk factors in the primary cohort.

### Outcomes

The outcome of our study was overall survival (OS). OS was defined as the time from the diagnosis of HCC to the date of the last follow-up or death. All GC patients were advised to receive regular follow-ups after completion of the primary therapy according to clinical guidelines. Patients who did not visit our hospital as scheduled were telephoned for follow-ups to obtain the treatment information and living status (performed by The Medical Information Unit in our Cancer Center). The last follow-up occurred in September 2018.

### Statistical analysis

Statistical analyses were performed using R for Windows (version 3.4.2, http://www.r-project.org/). A nomogram was formulated based on the results of multivariate analysis by the package of rms. We tested the accuracy of the nomograms by discrimination and calibration both in primary and externa validation cohort. The discrimination of the nomogram was measured by Harrell's C-index (C-index). The value of the C-index ranges from 0.5 to 1.0, with 0.5 indicating random chance and 1.0 indicating a perfect ability to correctly discriminate the outcome with the model. Then, the calibration curve of the nomogram model for the OS and decision curve analyses were performed. The total points of each patient were calculated according to the established Cox regression model, 3 groups of patients with different risk of prognosis (based on the total points) were delineated using the X-tile program 14 Survival curves were depicted by the Kaplan-Meier method, and using the dichotomized risk group as a factor, finally, compared using the log-rank test. All statistical tests were two-sided, and P values of less than 0.05 were considered to be statistically significant.

## Results

### Basic characteristics

In the development cohort, we included 836 consecutive LNM-positive GC patients, with 190(22.73%) patients died. While in the validation cohort, 411 patients were screened with 97(263.60%) patients died respectively. There are no significant differences between the two cohorts and basic characteristics are given in Table 1.

### Biomarker selection

**Figure 1:**
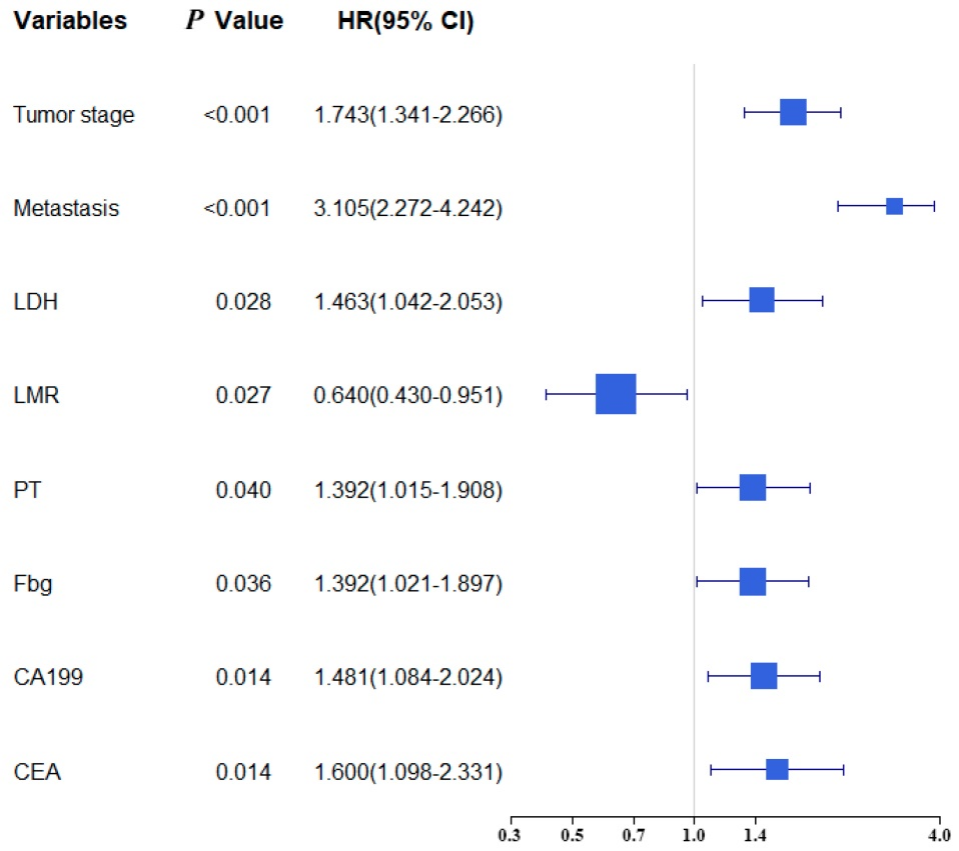
Forest plot showed the hazard ratio and 95% confidence interval for OS according to the Cox regression analysis.

Univariate and multivariate analysis were selected for evaluating the clinicopathologic characteristics and blood biomarkers (Table 2). The univariate analyse indicated age, TNM stage, Tumor stage, Metastasis, PLR, NLR, LMR, LDH, PT, APTT, TT, Fbg, CA199 and CEA were related to OS. In the multivariate analysis, eight of fourteen biomarkers were contained in final model (Tumor stage, Metastasis, LDH, LMR, Fbg, PT, CA199 and CEA). A forest plot shows the hazard ratios and 95% confidence intervals for OS according to the Cox proportional hazards regression analysis (Figure 1).

### Development and Validation of the Prediction Model

**Figure 2:**
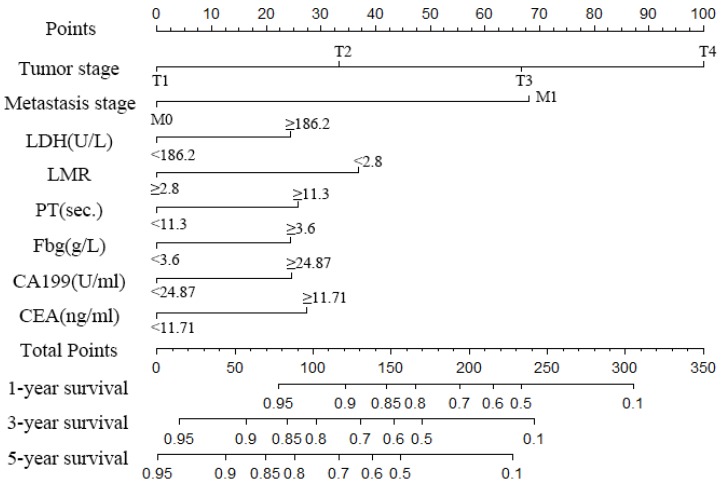
Nomogram to predict the probability of one-, three- and five-year overall survival (OS), including tumour stage, metastases stage, and LDH, LMR, PT, Fbg, CEA and CA199 levels in GC patients with LNM. The nomogram can be used to obtain the probability of one-, three- and five-year survival by adding up the points identified on the point scale for each variable.

**Figure 3:**
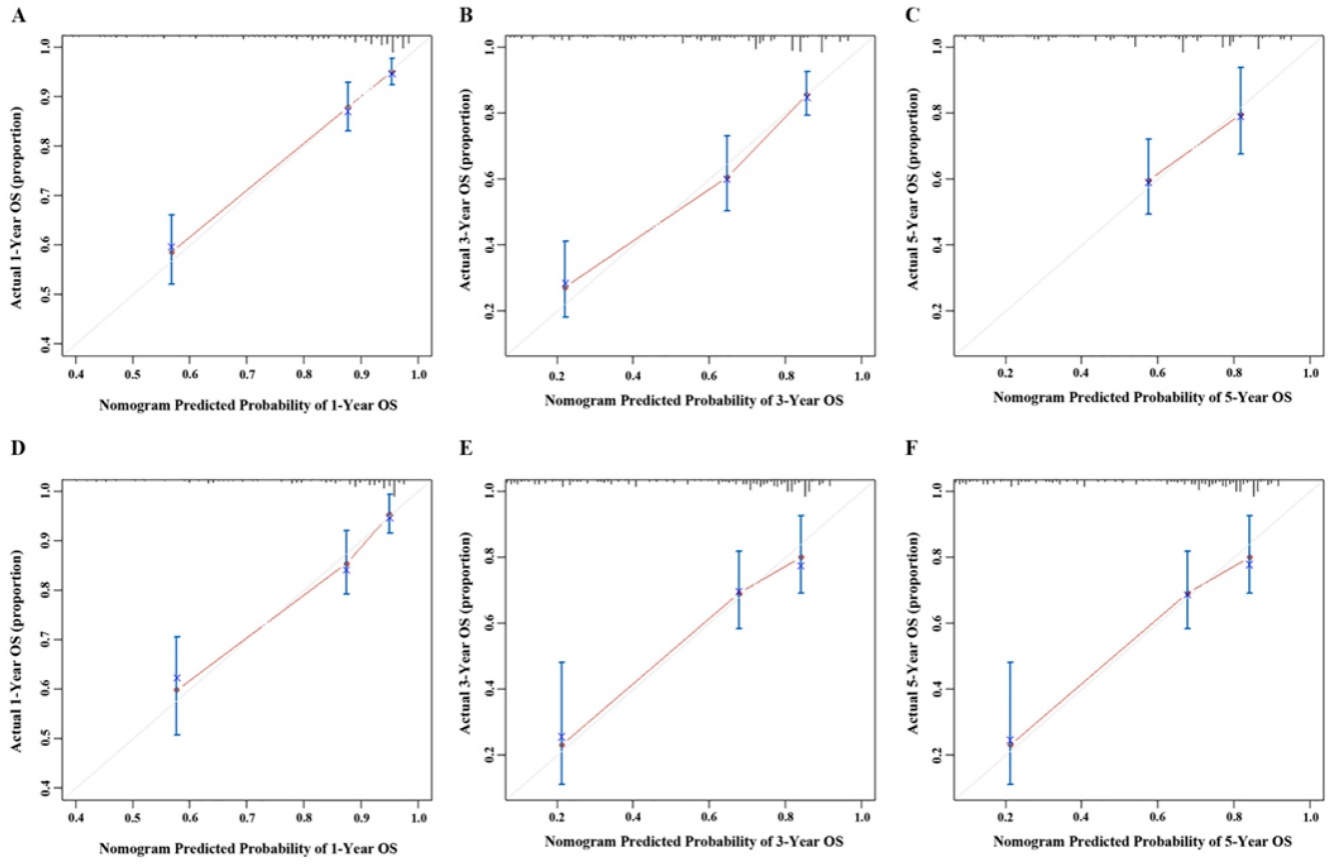
Calibration curve of the nomogram both in the primary and validation cohort. A. One-year OS in the primary cohort; B. Three-year survival OS in the primary cohort; C. Five-year survival OS in the primary cohort; D. One-year OS in the validation cohort; E. Three-year survival OS in the validation cohort; F. Five-year survival OS in the validation cohort.

A nomogram was created to predict the probability of a particular outcome. Figure 2 shows the nomogram predicting 1-, 3- and 5- year OS that was constructed that incorporated the above independent predictors. In the primary cohort, the C-index for OS prediction was 0.788 (95% CI: 0.753-0.823). Figure 3 showed that the calibration plot for the probability of OS at 1, 3 or 5 year after therapy demonstrated good agreement between the prediction by nomogram and actual observation. Furthermore, the discrimination of the nomogram have been compared with AJCC TNM Stage, the C-index of nomogram was 0.788 (95% CI: 0.753-0.823), which was superior to the C-index of AJCC TNM Stage 0.719 (95% CI: 0.687-0.751).

### Validation the Predictive Accuracy of Nomograms for OS

Validation was performed by using the other cohort of 411 LNM-positive GC patients. In the validation cohort, independent risk factors included in the nomogram were examined. Also, the C-index for OS prediction was up to 0.769 (95% CI: 0. 720-0.818). Figure 3 showed that the calibration plot for the probability of OS at 1, 3 or 5 year after therapy demonstrated good agreement between the prediction by nomogram and actual observation.

### Decision curve analysis

**Figure 4:**
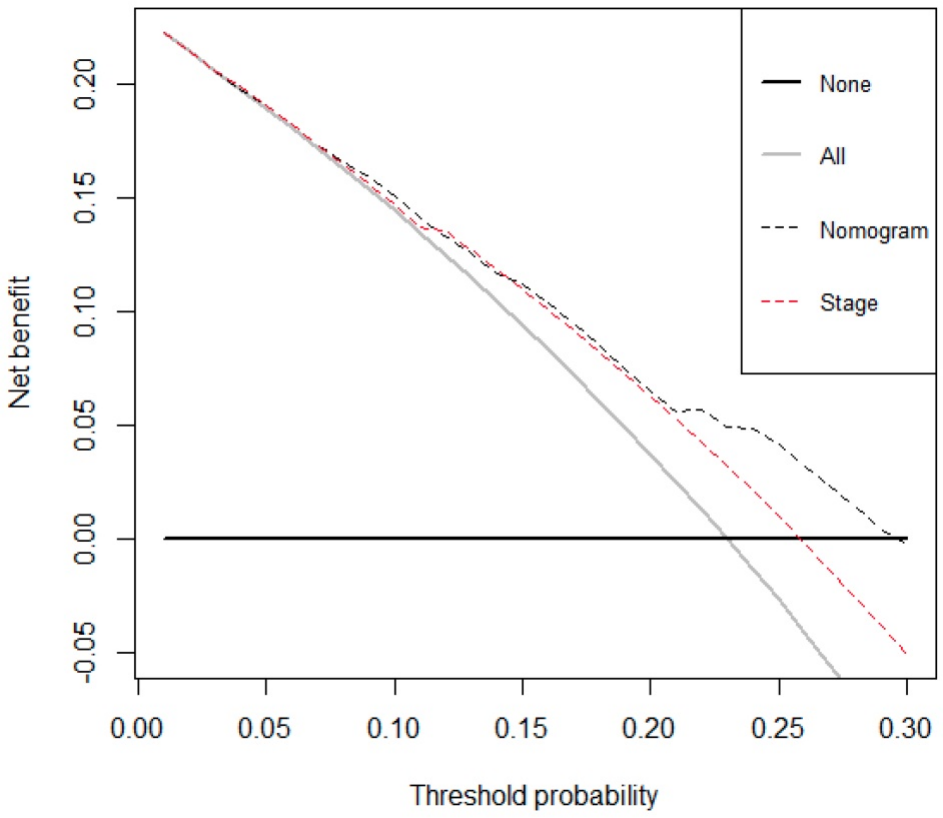
Decision curve analysis for overall survival. Black line: All patients died. Grey line: None of the patients died. Dashed black line: Nomogram model. Dashed red line: TNM staging system model.

The decision curve analysis for the nomogram and TNM staging systems is presented in Figure 4. The decision curve demonstrated that if the threshold probability of a patient is > 10%, the developed nomogram and TNM staging system in predicting OS is more benefit than all patients dead scheme or none patients dead scheme. Furthermore, the net benefit was comparable, the nomogram in predicting OS is more benefit than that of TNM staging system in this range.

### Risk stratification of OS by the nomogram model

**Figure 5:**
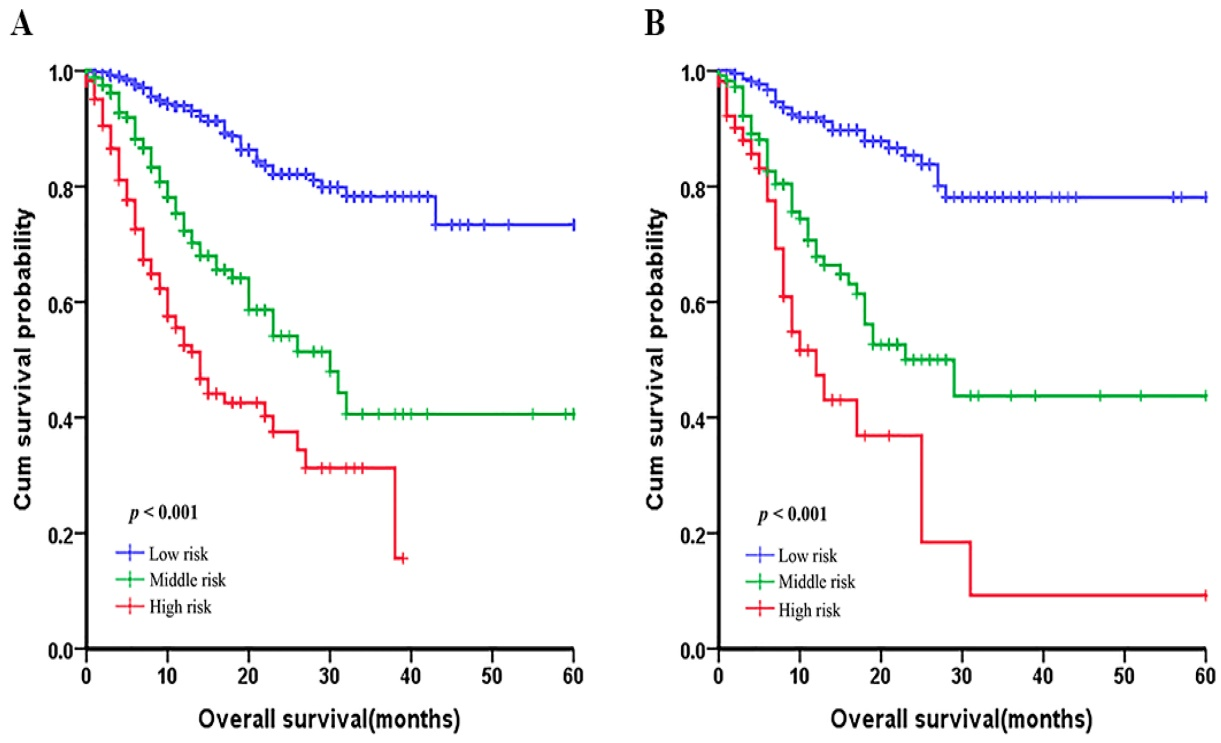
Graphs showing the Kaplan-Meier curves for all three groups based on the predictors from the nomogram model in the primary cohort (A) and those in the validation cohort (B).

In order to evaluate the subgroups of patients that were positively influenced by nomogram, we divided patients into three groups: low-risk group, middle-risk group and a high-risk group both in development cohort and validation cohort, which showed good prognostic classification for LNM-positive GC patients. In the primary cohort, the OS between the three risk groups were (17.35±11.90) months, (13.21±11.06) months, (7.25±6.54) months (p <0.001). Also, in the validation cohort, the mean OS between the three risk groups were (17.47±11.93) months, (14.30±11.23) months and (8.62±9.85) months (p <0.001) (Figure 5).

## Discussion

The present prognostic nomogram derived from prospectively collected data on 836 patients was shown to provide improved ability for individualized survival prediction in patients with LNM. By using this nomogram, individualization of patient counselling and decision-making regarding management can be improved.

As one of the most common malignant neoplasms in the digestive system, gastric cancer results in the death of thousands every year 15. Recurrence and metastasis is the major cause of GC treatment failure and death. Lymph node metastasis and organ invasion at diagnosis are the most important poor prognostic factors 16. In LNM-positive GC patients, the possibility to extend survival has been a topic of exploration for investigators globally, as improving life is always a consistent goal. Compared with LNM-negative patients, the overall recurrence rate in LNM-positive GC patients is obviously higher and the overall survival of LNM-positive GC patients is significantly shorter 17,18. Thus, the accurate tumor prognosis after definitive treatment is indispensable for LNM-positive GC patients.

Some studies have demonstrated that Systemic immune-inflammation index (SII) was developed and has proved to be a powerful prognostic indicator in hepatocellular carcinoma 19, and renal cell carcinoma patients 20, which based lymphocyte counts, neutrophil counts, and platelet counts. It is a close and complicated in peripheral blood where inflammation-based cells interact with tumor distant metastasis 5. The abnormal counts and ratio of neutrophil, lymphocyte, monocytes in the peripheral blood of cancer patients may be associated with tumor development 21. Platelet lymphocyte ratio (PLR) has been reported as an independent risk factor in gastric cancer 6,22. And it is reported that the coagulation pathway is essential for the establishment of metastasis in experimental model systems 23,24. Therefore, inflammatory markers in combination with other clinical characteristics may provide more reliable value for the LNM-positive GC patients.

At present, the TNM staging system as a tool to evaluate prognosis of patients who had gastric cancer, is current and accepted by commons, but it exists some limitations 25. The system only considers the anatomical extent of GC, ignoring the systemic immune-inflammation and coagulation heterogeneity of LNM-positive GC patients 26. Hence, we aimed to generate and validate a nomogram to overcome the above mentioned shorts of the TNM staging system as a means for survival prediction and treatment strategies guidance in patients with LNM. In our study, we found that T stage, M stage, LDH, LMR, PT, Fbg, CA199 and CEA were the factors that influenced prognosis of patients according to the multivariate analysis. Patients with an earlier stage of T stage, M stage, and a lower LDH, LMR, PT, Fbg, CA199 and CEA level have improved survival rates. Not only should we consider the impact of anatomical extent, but comprehensive systemic inflammatory biomarkers, metabolism of tumor, coagulation and traditional biomarkers, thus we developed more accurate prediction of the patient's prognosis.

Our nomogram is also a useful tool that utilizes conveniently available clinical information to provide simple prognostic information for oncologists and patients from complex statistical analysis. However, a major problem is to provide an accurate estimate of prognosis, especially, in patients with incurable cancers. Compared with the traditional TNM staging system, our method is more accurate and has a higher coincidence rate for patients with LNM. Our method combines other biomarkers including the systemic inflammatory biomarkers, coagulation, metabolism of tumor and traditional tumor biomarkers with the TNM stage, taking into account anatomy and LNM conditions, and more accurately predicts patients 1-year OS, 3- year OS, and 5-year OS. Simultaneously, the decision curve showed that the nomogram in predicting OS is better than that of TNM staging system in all range.

There are also some limitations in our study. First, lacking multi-center research data, the nomogram was created based on single data source and tested with only one external cohort in China. Second, there are no molecular or genetic biomarkers included in nomogram that have been reported to have independent prognostic value. Finally, in the validation cohort, the follow-up time was shorter, and patients in the validation cohort still needed close monitoring and five-year follow-up data. In addition, future research can incorporate the LNM-positive GC patient's quality of life into the research system, and the nutritional status and quality of life of LNM-positive GC patients during the survival period have the same important status as the prolonged survival time. Despite these limitations, this nomogram represents a prognostic effect on patients with LNM-positive GC. We anticipate that this nomogram will stimulate ongoing research and multiple-center clinical research with wider geographic recruitment will further improve and validate it.

## Conclusions

The present nomogram can predict the prognosis of LNM-positive GC patients, potentially facilitating highly tailored patient management. This nomogram represents an improvement in prognostication over the current TNM stage.

## Figures and Tables

**Table 1:** Baseline clinical features

	Development cohort (n=836)	Validation Cohort(n=411)
Characteristics	Mean±SD/ No(%)	Mean±SD/ No(%)
Age, year	57.10±11.94	57.89±11.58
Sex		
Male	527(63.04%)	272(66.18%)
Female	309(36.96%)	139(33.82%)
TNM stage		
Ⅰ	17(2.03%)	5 (1.22%)
Ⅱ	99(11.84%)	45(10.95%)
Ⅲ	453(54.19%)	218(53.04%)
Ⅳ	267(31.94%)	143(34.79%)
Tumor stage		
T1	37(4.43%)	13 (3.16%)
T2	49(5.86%)	20(4.87%)
T3	308(36.84%)	151(36.74%)
T4	442(52.87%)	227(55.23%)
Metastasis		
No	569(68.06%)	268(65.21%)
Yes	267(31.94%)	143(34.79%)
Lauren		
1	270(32.30%)	134(32.60%)
2	345(41.27%)	155(37.71%)
3	221(26.44%)	122(29.68%)
PLR		
≤290.8	729(87.20%)	362(88.08%)
>290.8	107(12.80%)	49(11.92%)
NLR		
≤2.8	513(61.36%)	256(62.29%)
>2.8	323(38.63%)	155(37.71%)
LMR		
≤2.8	220(26.31%)	116(28.22%)
>2.8	616(73.68%)	295(71.78%)
LDH		
≤186.2	145.12±21.22	148.01±21.16
>186.2	255.51±241.22	245.99±90.26
PT, S		
≤11.3	10.69±0.39	10.65±0.40
>11.3	12.11±1.00	11.97±0.60
APTT		
≤31.2	25.45±2.94	25.47±2.84
>31.2	34.16±3.20	33.25±1.68
TT		
≤18.0	16.98±0.71	16.94±0.79
>18.0	19.20±1.34	19.13±1.20
Fbg,g/L		
≤3.6	2.79±0.52	2.74±0.53
>3.6	4.39±0.65	4.36±0.63
CEA		
≤11.71	2.73±2.11	2.84±2.14
>11.71	116.45±259.56	256.67±929.68
CA199		
≤24.87	9.56±5.82	10.01±6.27
>24.87	523.52±1775.40	467.01±1347.50

Data are presented as mean (SD) or N (%).

**Table 2:** Univariate and multivariate cox hazards analysis between clinical features and OS

	Univariate analysis		Multivariate analysis	
Characteristics	HR (95% CI)	P-value	HR (95% CI)	P-value
Age, year	1.437(1.076-1.919)	0.014	1.339(0.988-1.815)	0.060
Sex				
Male/Female	0.892(0.762-1.367)	0.892		
TNM stage				
Ⅰ/Ⅱ/Ⅲ/Ⅳ	3.537(2.751-4.547)	<0.001		
Tumor stage				
T1/ T2/ T3/ T4	2.040(1.601-2.599)	<0.001	1.743(1.341-2.266)	<0.001
Metastasis				
No/ Yes	4.271(3.199-5.702	<0.001	3.105(2.272-4.242)	<0.001
Lauren				
1/2/3	1.006(0.836-1.211)	0.952		
PLR				
≤290.8/>290.8	2.118(1.494-3.003)	<0.001	1.432(0.935-2.194)	0.099
NLR				
≤2.8/>2.8	2.092(1.573-2.783)	<0.001	1.007(0.699-1.449)	0.972
LMR				
≤2.8/>2.8	0.402(0.300-0.538)	<0.001	0.640(0.430-0.951)	0.027
LDH				
≤26.9/>26.9	1.885(1.382-2.571)	<0.001	1.463(1.042-2.053)	0.028
PT, S				
≤11.9/>11.9	1.770(1.321-2.373)	<0.001	1.392(1.015-1.908)	0.040
APTT				
≤11.9/>11.9	1.570(1.036-2.379)	0.033	1.297(0.830-2.027)	0.253
TT				
≤11.9/>11.9	0.535(0.398-0.720)	<0.001	0.836(0.609-1.148)	0.269
Fbg,g/L				
≤3.6/>3.6	2.050(1.525-2.757)	<0.001	1.392(1.021-1.897)	0.036
CEA				
≤11.71/>11.71	2.688(1.926-3.752)	<0.001	1.600(1.098-2.331)	0.014
CA199				
≤24.87/>24.87	2.416(1.798-3.246)	<0.001	1.481(1.084-2.024)	0.014

## Abbreviations

GC: gastric cancer
LNM: lymph node metastasis
OS: Overall Survival
SII: systemic immune-inflammation index
TNM: tumor, lymph node and metastases
DIC: disseminated intravascular coagulation
PLR: platelet-lymphocyte ratio
NLR: rationeutrophil-lymphocyte ratio
LMR: lymphocyte-mononuclyte ratio
LDH: lactate dehydrogenas
PT: Prothrombin time
APTT: activated partial thromboplastin time
TT: thrombin time
Fbg: fibrinogen
CEA: carcinoembryonic antigen
CA199: carbohydrate antigen 19-9
